# Influenza Aerosols in UK Hospitals during the H1N1 (2009) Pandemic – The Risk of Aerosol Generation during Medical Procedures

**DOI:** 10.1371/journal.pone.0056278

**Published:** 2013-02-13

**Authors:** Katy-Anne Thompson, John V. Pappachan, Allan M. Bennett, Himanshu Mittal, Susan Macken, Brian K. Dove, Jonathan S. Nguyen-Van-Tam, Vicky R. Copley, Sarah O’Brien, Peter Hoffman, Simon Parks, Andrew Bentley, Barbara Isalska, Gail Thomson, EASE Study Consortium

**Affiliations:** 1 Biosafety Investigation Unit, Health Protection Agency, Porton Down, Wiltshire, United Kingdom; 2 Paediatric Intensive Care Unit, University Hospital Southampton NHS Foundation Trust, Southampton, Hampshire, United Kingdom; 3 Biosafety Investigation Unit, Health Protection Agency, Colindale, London, United Kingdom; 4 Influenza Group, Health Protection Agency, Porton Down, Wiltshire, United Kingdom; 5 Health Protection and Influenza Research Group, University of Nottingham, Nottingham, United Kingdom; 6 Microbial Risk Assessment Group, Health Protection Agency, Porton Down, Wiltshire, United Kingdom; 7 Institute of Infection and Global Health, University of Liverpool, Liverpool, United Kingdom; 8 Antimicrobial Resistance and Healthcare-Associated Reference Unit, Health Protection Agency, Colindale, London, United Kingdom; 9 Acute Intensive Care Unit, University Hospital of South Manchester, Wythenshawe, Manchester, United Kingdom; 10 Department of Microbiology, University Hospital of South Manchester, Wythenshawe, Manchester, United Kingdom; 11 Medical Affairs, Health Protection Agency, Porton Down, Wiltshire, United Kingdom; University of Calgary & ProvLab Alberta, Canada

## Abstract

**Background:**

Nosocomial infection of health-care workers (HCWs) during outbreaks of respiratory infections (e.g. Influenza A H1N1 (2009)) is a significant concern for public health policy makers. World Health Organization (WHO)-defined ‘aerosol generating procedures’ (AGPs) are thought to increase the risk of aerosol transmission to HCWs, but there are presently insufficient data to quantify risk accurately or establish a hierarchy of risk-prone procedures.

**Methodology/Principal Findings:**

This study measured the amount of H1N1 (2009) RNA in aerosols in the vicinity of H1N1 positive patients undergoing AGPs to help quantify the potential risk of transmission to HCWs. There were 99 sampling occasions (windows) producing a total of 198 May stages for analysis in the size ranges 0.86–7.3 µm. Considering stages 2 (4–7.3 µm) and 3 (0.86–4 µm) as comprising one sample, viral RNA was detected in 14 (14.1%) air samples from 10 (25.6%) patients. Twenty three air samples were collected while potential AGPs were being performed of which 6 (26.1%) contained viral RNA; in contrast, 76 May samples were collected when no WHO 2009 defined AGP was being performed of which 8 (10.5%) contained viral RNA (unadjusted OR = 2.84 (95% CI 1.11–7.24) adjusted OR = 4.31 (0.83–22.5)).

**Conclusions/Significance:**

With our small sample size we found that AGPs do not significantly increase the probability of sampling an H1N1 (2009) positive aerosol (OR (95% CI) = 4.31 (0.83–22.5). Although the probability of detecting positive H1N1 (2009) positive aerosols when performing various AGPs on intensive care patients above the baseline rate (i.e. in the absence of AGPs) did not reach significance, there was a trend towards hierarchy of AGPs, placing bronchoscopy and respiratory and airway suctioning above baseline (background) values. Further, larger studies are required but these preliminary findings may be of benefit to infection control teams.

## Introduction

In 2003, at least 284 healthcare workers (HCWs) were infected with SARS-Coronavirus during the severe acute respiratory syndrome epidemic [1]. Nosocomial infection was the primary accelerator of infection accounting for 72% of cases in Toronto and 55% of probable cases in Taiwan [2], [3]. Aerosol generating procedures (AGPs) performed on infected patients were implicated as the source of outbreaks among Health Care Workers (HCWs), however no direct evidence for this mode of transmission was demonstrated. [4].

Several medical procedures have been reported to generate aerosols and to increase the risk of pathogen transmission [5]. In response to these concerns the World Health Organization (WHO) produced guidelines on infection control procedures and personal protective equipment in 2007, which were refined in 2009 (Table 1) [6], [7]. The guidelines incorporated the available data relating to the infective potential of AGPs but many of the studies contained methodological flaws that precluded the use of their conclusions to draw recommendations, and it was accepted that the level of understanding of the aerobiology of AGPs may evolve. In particular the risks associated with individual AGPs have not been quantified nor has a risk hierarchy been established.

**Table 1:** In December 2009 the World Health Organization (WHO) updated its advice on AGPs.

2007 WHO Guidance	2009 WHO Guidance
Intubation, and related procedures (e.g. manual ventilation, suction)	Intubation and related procedures, e.g. manual ventilation
Cardiopulmonary resuscitation	Cardiopulmonary resuscitation
Bronchoscopy	Bronchoscopy
Autopsy/surgery	Autopsy procedures
Non-invasive positive pressure ventilation and bilevel positive airway pressure	Respiratory and airway suctioning (including tracheostomy care and open suctioning with invasive ventilation)
High-frequency oscillating ventilation	Collection of lower respiratory tract specimens (e.g. bronchial and tracheal aspirates)
Nebulisation	

N.B. Chest physiotherapy is now not considered an AGP but advice states that a surgical mask should be worn by the patient if tolerated and HCWs should wear PPE as recommended for routine care (i.e. a surgical mask) during the procedure.

Despite their scientific limitations these guidelines have been used to plan infection control during subsequent pandemics. In addition, controversy remains about the importance of aerosol transmission of influenza in the absence of AGPs [8], [9], [10]. This ongoing uncertainty was reflected in the diverse approaches adopted by different countries in relation to the recommendations for the use of surgical face masks (SFMs) and respirators by HCWs during the 2009 pandemic [11], [12], [13].

WHO guidance states that standard and droplet precautions (i.e. SFMs) should be adopted when working in direct contact with infected patients and high level respiratory protective equipment (minimum of an FFP2/N95 class respirator) should be worn only in the vicinity of infected patients when AGPs are performed [5], [6]. As a result, this infection control strategy was adopted in all U.K. NHS organisations [14].

Infectious pathogens may be contained in aerosols which are generally recognised to be <5 µm in aerodynamic diameter, remain suspended for periods of time and travel significant distances. Safety in the presence of infectious aerosols requires respiratory protective equipment (UK standard - FFP3 respirators) to protect exposed HCWs [14]. During human influenza infection, virus emission occurs via coughing and sneezing, which produce a ‘respiratory spray’ containing particles in a size continuum from <1 to >500 µm [15].

The initial outbreak and subsequent worldwide spread of influenza A (H1N1) 2009 provided an opportunity to perform air sampling to define the viral RNA copy number, and size of aerosols that an AGP (defined by WHO) produces and the relative burden compared to freely respiring patients.

## Methods

### Objectives

The primary objectives of this study were to:

1. Establish if World Health Organization defined ‘aerosol generating procedures’ produce infectious aerosols.
2. If detectable clouds are produced then determine infectious aerosol concentration and particle size.
3. To use this information to inform infection control practice.

### Participants

**Figure 1:**
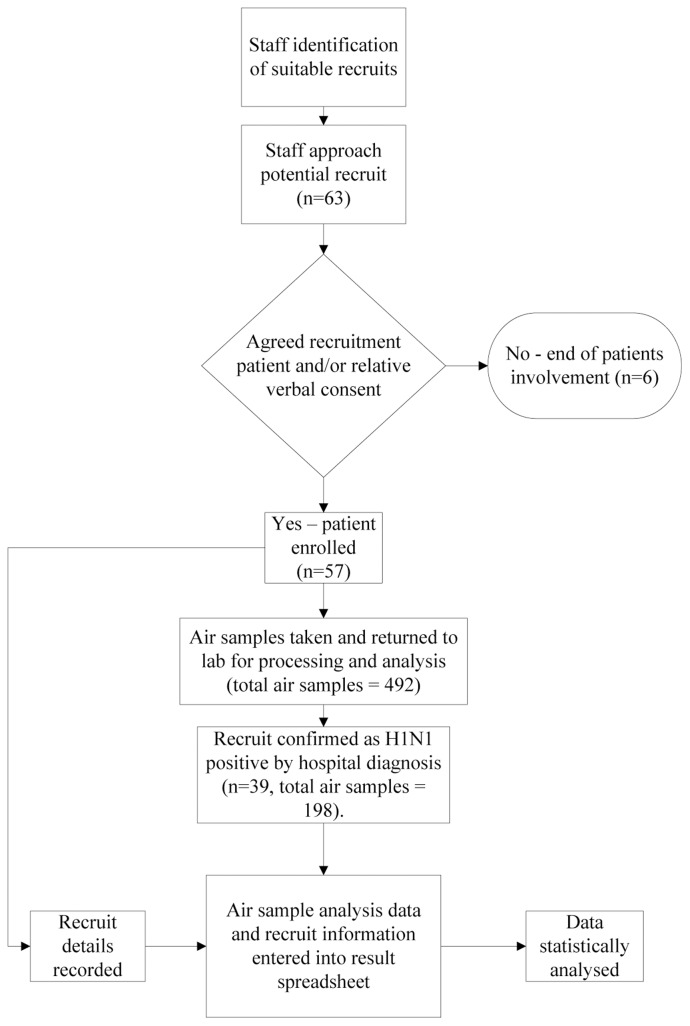
The recruitment and data analysis flowchart.

Air was sampled around hospitalised patients with suspected or proven lower respiratory tract infection located either in pandemic H1N1 (2009) isolation rooms (single occupancy rooms) or cohort areas (wards in which numerous patients suspected or confirmed as being H1N1 positive were placed away from other patients). Sampling was performed in 5 different hospitals located around England, U.K. This study was performed from October 2009 to January 2011. Peak sampling times corresponded to known periods of heightened H1N1 (2009) activity. Potential recruits were identified in participating units by local principal investigators who informed the sampling team. The sampling team was deployed to units with the highest level of overall activity. Patient inclusion criteria were defined as new chest X-Ray changes (e.g. consolidation, alveolar infiltrates or atelectasis) in the presence of one or more of the following: central temperature ≥38°C (38.5°C in children) or ≤36°C, white blood cell count <4000 cells/mm^2^ or >12,000 cells/mm^2^, positive microbiology or virology from respiratory secretions and mucopurulent secretions from the respiratory tract (Figure 1). During air sampling, investigators recorded any clinical interventions performed, as well as temperature, relative humidity, time and location of air sample, activity levels, patient sex, age, diagnostic specimen type, results and time since admission. Using these data, two consultant physicians (JP & MB), blinded to the air sampling data and the H1N1 status of the patients, independently coded interventions as AGPs if they met 2007 and 2009 WHO definitions (Table 1). Any disagreements were discussed until a consensus was reached. Baseline samples were those taken during interventions not meeting the WHO definitions of an AGP or taken at least 30 minutes after a WHO defined AGP had been completed. All recruits had at least one baseline sampling event, but incidence of WHO defined AGPs was rarer and investigators attempted to capture as many of these as possible.

### Description of Procedures or Investigations Undertaken

Glass May 3-stage impingers (produced at HPA, Porton Down), operating at 55 litres per minute and placed at 1 meter height within 1 meter of the patients head, were used to collect air samples for a maximum of 40 minutes [16]. Baseline samples were taken for 10 minutes. Air was collected into 15 mls of sterile, nuclease free, molecular grade, phosphate buffered saline. The May 3-stage impinger fractionates air particles into 3 aerodynamic size ranges, stage 1 collects particles >7.3 µm, stage 2 collects particles in the range 4–7.3 µm and stage 3 collects particles 0.86–4 µm. Liquid air samples were transported to the laboratory frozen on dry ice and stored at −20°C until analysis.

The sampling team and all staff/visitors were wearing FFP3 respirators in cohort areas and isolation rooms during the 2009/2010 pandemic. In addition, the sampling team, and a proportion of the staff/visitors were vaccinated against the pandemic strain and the majority of other patients in the intensive care units were on closed ventilator circuits. This means that it is unlikely that the influenza aerosols could have been generated by anyone other than the patient on whom the AGP was being performed. In the case of baseline samples there was often some form of non-AGP classified activity ongoing (e.g. activity associated with chest physiotherapy or respiratory care), and as such these samples do not represent ‘control’ samples where no activity was taking place, they merely represent air samples taken when no activity that could be defined as an AGP was taking place.

### Sample Processing and Analysis Methodology

On arrival at the laboratory samples were stored at −20°C prior to RNA extraction. Each sample was concentrated into <500 µl using 30 kD macrosep centrifugal devices (5000 g for 1.5 hours at 4°C). Any viral RNA present within the resulting concentrate was extracted using the Qiagen QIAamp viral RNA spin protocol (Qiagen, U.K.) and eluted into 2×30 µl volumes of elution buffer which were subsequently pooled. Samples were frozen at −20°C and defrosted prior to use in the qRT-PCR assay.

A pan-influenza A qRT-PCR assay, based on the pan-influenza segment 7 assay developed by Spackman *et al*. (2002), was used to detect the presence of any influenza A RNA within the samples [17]. The primer, probe concentrations and RNA template volumes were re-optimised from the conditions originally described. The hydrolysis probe was also adapted to use a black hole quencher (BHQ) rather than Carboxytetramethylrhodamine (TAMRA) increasing assay sensitivity. A standard curve was also added to the assay to allow quantification of influenza RNA within the samples. This was prepared using 2009 pandemic nH1N1 A/Cal/04/09 influenza virus. RNA extracted from a live virus preparation was performed using the QIAamp viral RNA mini kit (Qiagen). The full-length influenza segment 7 was amplified by RT-PCR using RT primer Uni12 and PCR primers Bm-M-1and BM-M-1027 R as described in Hoffman *et al*. (2001) [17]. The amplicon was purified from a 1% (w/v) agarose gel using the QIAquick gel extraction kit (Qiagen), cloned into pCR 2.1 TOPO vector and heat-shocked into *E. Coli* TOP10 cells, as described in the TOPO TA cloning kit (Invitrogen), to construct pCR 2.1 TOPO-CalM. The influenza A/Cal/04/09 segment 7 insert within pCR 2.1 TOPO-CalM was confirmed by sequencing using M13 forward and reverse priming sites that flanked the insert (data not shown). The H1N1 A/Cal/04/09 segment 7 sequence was then amplified from pCR2.1 TOPO-CalM by PCR using primers MCalFwd1 (5′-AGCTAGGCATGCGCGGCCGCAGCAAAAGCAGGTAG-3′) and MCalRev1 (5′-AGCTAGGCATGC*TAATACGACTCACTATAGGG*AGTAGAAACAAGGTAGTTTTT-3′) which contained a T7 RNA polymerase promoter sequence (highlighted in italics). A negative sense T7 RNA transcript of the Cal/04/09 M segment sequence was generated from the PCR amplicon, with the amplicon subsequently removed by DNase digestion, using the Megascript T7 kit (Ambion). DNAse digestion of the amplicon template was confirmed by agarose get electrophoresis. The RNA transcript was purified using an RNeasy mini kit (Qiagen) RNA clean-up protocol and the copy number calculated following determination of the RNA transcript concentration with a Nanodrop ND100 spectrophotmeter.

All qRT-PCR reactions were performed on 96-well plates using the superscript III platinum one-step quantitative RT-PCR kit (Invitrogen) and run on the Applied Biosystems 7900 real-time PCR system. Each 96-well plate consisted of a standard curve (prepared from a ten-fold dilution series of the nH1N1 A/Cal/04/09 M segment RNA transcript in H_2_O) which also acted as the assay positive control, a non-template negative control (molecular grade water) and RNA from processed air samples. All controls and samples were run in duplicate. qRT-PCR reactions consisted of 12.5 µl 2×Buffer, 1.25 µl M+25 oligo (AGA TGA GTC TTC TAA CCG AGG TCG) (300 nM final concentration), 1.25 µl M-125 oligo (TGC AAA AAC ATC TTC AAG TCT CTG) (300 nM final concentration), 0.5 µl Rox, 0.5 µl Taq/superscript enyzme, 0.25 µl M+65 probe (FAM-TCA GGC CCC CTC AAA GCC GA-BHQ) (250 nM final concentration) and 8.75 µl of either sample RNA, standard curve RNA or negative control (dH_2_O) to reach a final volume of 25 µl. The Influenza qRT-PCR Cycling Conditions were 50°C for 30 minutes, 95°C for 10 minutes, followed by 50 cycles of 95°C for 15 seconds and 60°C for 60 seconds. The number of virus copies/litre were for each sample was then determined using the standard curve (R^2^ values were typically 0.94).

### Ethics

The study was approved by Oxfordshire Local Research Ethics Committee (REC reference 09/H0604/119) and the requirement for informed consent was waived. The committee agreed that this study did not constitute intrusive research because it involved only the processing of non-identifiable data. The committee felt that the provisions of the Mental Capacity Act did not apply to this study. The committee welcomed the statements by the researchers that they would always seek the views of patients, parents and/or relatives prior to starting to take air samples and that they would respond to concerns after the sampling had started. The committee is constituted in accordance with the Governance Arrangements for Research Ethics Committees (July 2001) and complies fully with the Standard Operating Procedures for Research Ethics Committees in the U.K.

### Statistical Methods

Statistical analysis was performed with Stata software (version 11.0; Stata Corp., College Station, TX, USA). Only patients confirmed as influenza A H1N1 (2009) RT-PCR positive from respiratory tract samples were considered in the statistical analysis. Univariable logistic regression models were used to examine the relationship between propensity to produce a positive H1N1 aerosol sample and a number of potential risk factors. Wald tests were used to assess risk factor significance. A sample was considered to be positive for aerosolised influenza particles if either or both of May stages 2 and 3 (0.86–7.3 µm) indicated the presence of H1N1. Copy numbers from stages 2 and 3 were summed to give a total copy number for the sampling occasion. A random effect was included in the logistic models at subject level to account for potential correlation caused by repeated measurements on the same individual. In instances where a positive H1N1 sample was produced, univariable negative binomial regression models, corrected for sample duration, were used to examine the relationship between total copy number per litre per minute and potential risk factors. Only three participants gave more than one positive H1N1 sample and so a random subject-level effect was not used in the negative binomial regressions. Instead a robust standard error was used to adjust for potential correlation in these models.

Finally, the probability of obtaining a positive sample and the viral load obtained from positive samples were modelled to rank the 2007 and 2009 WHO procedure lists and chest physiotherapy and produce a hierarchy of procedures on the basis of potential risk. The classical definition of risk as equal to ‘probability times consequence’ was used and hence multiplied predictions from the logistic and negative binomial models together in order to obtain a risk measure which gives the expected value of copy number (l/min) associated with each procedure.

## Results

A total of 57 patients were studied, however only 39 patients were later confirmed as influenza A H1N1 (2009) RT-PCR positive and were included in the statistical analysis.

Table 2 and 3 show the percentage of total RNA found in each stage of the May impinger (Table 2) and the % of RNA collected in each stage during each procedure (Table 3). These tables show that most of the RNA recovered from the baseline samples has been recovered in the >7.3 µm size range (53.6% of total RNA and 78.7% of the total RNA recovered from baseline samples). In contrast, the total amount of RNA recovered from all the bronchoscopy samples was only 7.1% (confounded by limited sample size) but 75.1% of this was collected in the stages <7.3 µm. The situation is similar for respiratory and airway suctioning (3.0% total RNA of which 77.6% was in the <7.3 µm size range). Therefore the results indicate that AGPs as defined by the WHO 2009 definitions tend to produce aerosols of smaller particle sizes than baseline levels.

**Table 2:** The percentage of total RNA collected in each stage (size range) of the May impinger compared based on the WHO 2009 AGP definitions.

Procedure	% RNA collected >7.3 µm	% RNA collected 4–7.3 µm	% RNA collected 0.86–4 µm
Baseline	53.6	7.6	6.9
Bronchoscopy	7.1	12.9	8.6
Respiratory &Airway Suction	0.7	0.9	1.4
Intubation	0.0	0.3	0.0

**Table 3:** The % of RNA collected from each procedure in each stage of May sampler.

Procedure	Number of sampling occasions (number of patients)	% RNA collected >7.3 µm	% RNA collected 4–7.3 µm	% RNA collected 0.86–4 µm	Median copy no./l (inter-quartile range) for samples with at least one stage with detectable RNA
Baseline	76 (39)	78.7	11.1	10.2	7,913 (2,436–11,613)
Bronchoscopy	3 (3)	24.9	45.2	29.9	148,805 (12,735–284,875)
Respiratory &Airway Suction	14 (11)	22.4	29.7	47.9	1,852 (1,543–2,7521)
Intubation	5 (4)	0.0	100.0	0.0	2,838 (2,838–2,838)

Figure 1 shows the patient inclusion process but as this paper is interested in the ability of AGPs to generate aerosols rather than respiratory droplets subsequent analysis deals with the results from the second and third stages (particle sizes 0.86–7.3 µm) of the May sampler, however full results can be seen as an online supplement. Tables 4 & 5 show H1N1 patient demographics together with other characteristics of their samples.

**Table 4:** Location and Environmental conditions of air samples.

Characteristic	H1N1 positive patients (n = 39)			H1N1 positive aerosol samples^a^ (n = 14; collected from 10 patients)			A		B	
	n	%	mean (sd)	n (n unique patients^b^)	%	Median copy no./l (inter- quartile range)	OR (95% CI)	p value	IRR (95% CI)	p value
Centre ID										
Location 1	19	49		4 (3)	29	8,888 (4,611–10,738)	referent		referent	
Location 2	10	26		8 (5)	57	7,741 (1,698–62,343)	5.86 (1.10–31.37)		6.21 (2.49–15.5)	
Location 3	6	15		0	0		–		–	
Location 4	3	8		2 (2)	14	6,292 (2,838–9,745)	3.70 (0.37–37.55)		0.38 (0.28–0.52)	
Location 5	1	3		0	0		–	0.114	–	<0.001
Location										
Cohort area	24	62		11 (9)	79	7,913 (1,852–9,963)	referent		referent	
Isolation	15	38		3 (1)	21	20,614 (1,543–213,659)	0.27 (0.03–2.20)	0.22	5.20 (0·79–34.3)	0.087
Air sample volume (l)							1.00 (0.999–1.004)	0.137	0.998 (0.998–0.999)	<0.001
Temperature °C			22.2 (1.42)				1.21 (0.70–2.08)	0.49	1.07 (0.27–4.16)	0.923
Relative humidity			33.6 (11.12)				0.95 (0.89–1.02)	0.154	0.95 (0.91–0.98)	0.006

a Sample considered positive if aerosol <7.3 µm indicated the present of H1N1.

b n unique patients refers to the number of patients these samples were taken from.

Column A: Results from univariable logistic regression models examining potential risk factors for production of H1N1 positive aerosol. Sample considered positive if aerosol <7.3 µm indicated the presence of H1N1. Estimates adjusted for repeated measurements.

Column B: Results from univariable negative binomial regression models examining potential risk factors for copy number per litre per minute given H1N1 positive aerosol.

A hyphen (-) indicates no positive samples in category. Referent refers to the category of an ordinal or nominal variable against which other categories are compared in the regression model and for which *no* dummy variable is included in the regression model.

**Table 5:** Demographic and patient specific conditions of air samples.

Characteristic	H1N1 positive patients (n = 39)			H1N1 positive aerosol samples^a^ (n = 14; collected from 10 patients)			A		B	
	n	%	mean (sd)	n (n unique patients^b^)	%	Median copy no./l(inter- quartile range)	OR (95% CI)	p value	IRR (95% CI)	p value
Sex										
Male	25	64		11 (7)	79	9,745 (1,543–11,613)	referent		referent	
Female	14	36		3 (3)	21	5,518 (2,838–104,072)	0.45 (0.09–2.33)	0.342	1.57 (0.22–11.3)	0.651
Age										
<5	4	10		0	0		–		–	
5–50	7	18		4 (3)	29	4,883 (1,262–8,938)	2.62 (0.37–18.8)		0.71 (0.41–1.25)	
50–60	13	33		6 (4)	43	15,180 (5,518–104,072)	2.43 (0.43–13.7)	0.525	8.85 (4.14–18.9)	<0.001
60–70+	15	39		4 (3)	29	6,350 (2,073–10,738)	referent		referent	
ECMO										
No	34	87		10 (8)	71	8,829 (2,838–9,963)	referent		referent	
Yes	5	13		4 (2)	29	11,233 (1,698–117,137)	2.88 (0.44–18.9)	0.269	3.62 (0.83–15.8)	0.087
Repeats per patient			2.5 (1.27)							
Days since diagnosis			7.5 (8.56)				0.96 (0.87–1.06)	0.422	0.84 (0.79–0.90)	<0.001
Days since last positive sample			5.5 (6.32)				1.02 (0.91–1.15)	0.719	0.84 (0.78–0.90)	<0.001
Repeat number			7.5 (8.56)				1.22 (0.70–2.11)	0.483	2.36 (0.91–6.14)	0.078

a Sample considered positive if aerosol <7.3 µm indicated the present of H1N1.

b n unique patients refers to the number of patients these samples were taken from.

Column A: Results from univariable logistic regression models examining potential risk factors for production of H1N1 positive aerosol. Sample considered positive if aerosol <7.3 µm indicated the presence of H1N1. Estimates adjusted for repeated measurements.

Column B: Results from univariable negative binomial regression models examining potential risk factors for copy number per litre per minute given H1N1 positive aerosol.

A hyphen (-) indicates no positive samples in category. Referent refers to the category of an ordinal or nominal variable against which other categories are compared in the regression model and for which *no* dummy variable is included in the regression model.

There were 99 sampling occasions (windows) producing a total of 198 May stages for analysis in the size ranges 0.86–7.3 µm (Table 6). Forty-six air stage samples were collected while potential AGPs (according to WHO 2009 definitions – see Table 1) were being performed of which 9 (19.6%) contained viral RNA; in contrast, 152 May stage samples were collected when no WHO 2009 defined AGP was being performed of which 12 (7.9%) contained viral RNA (unadjusted OR = 2.84 (95% CI: 1.11–7.24) adjusted OR = 4.31 (0.83–22.5)).

**Table 6:** Analysis of air samples using the WHO (2009) AGP definitions.

Characteristic	All repeats (n = 99)		H1N1 positive aerosol samples^a^ (n = 14; collected from10 patients)			A		B	
	n	%	n (n unique patients^b^)	%	Median copy no./l (inter- quartile range)	OR (95% CI)	p value	IRR (95% CI)	p value
AGP (2009)									
Baseline (no AGP)	76	77	8 (6)	57	8,888 (3,414–10,788)	referent		referent	
Bronchoscopy	3	3	2 (2)	14	111,702 (9,745–213,659)	36.8 (0.96–1409)		5.41 (0.77–38.0)	
Lower respiratory tract specimen collection	1	1	0	0		–		–	
Intubation & related procedures	5	5	1 (1)	7	2,838 (2,838–2,838)	2.37 (0.13–41.5)		0.15 (0.04–0.58)	
Respiratory/airway suction	14	14	3 (2)	22	1,852 (1,543–20,614)	3.53 (0.45–27.5)	0.231	0.43 (0.09–1.99)	not available
AGP (2009) defined									
No						referent		referent	
Yes						4.31 (0.83–22.5)	0.083	2.04 (0.36–11.62)	0.42

a Sample considered positive if aerosol <7.3 µm indicated the present of H1N1.

b n unique patients refers to the number of patients these samples were taken from.

Column A: Results from univariable logistic regression models examining potential risk factors for production of H1N1 positive aerosol. Sample considered positive if aerosol <7.3 µm indicated the presence of H1N1. Estimates adjusted for repeated measurements.

Column B: Results from univariable negative binomial regression models examining potential risk factors for copy number per litre per minute given H1N1 positive aerosol.

A hyphen (-) indicates no positive samples in category. Referent refers to the category of an ordinal or nominal variable against which other categories are compared in the regression model and for which *no* dummy variable is included in the regression model.

There were 99 May stages for analysis in the >7.3 µm size range, 23 air stage samples were collected while potential WHO (2009) AGPs were being performed of which 3 (13.0%) contained viral RNA; 76 May stage samples were collected when no WHO (2009) AGP was being performed of which 9 (11.8%) contained viral RNA. This was not statistically significant (unadjusted OR = 1.06 (0.26–4.29)).

Results from the logistic regressions (Tables 4, 5, 6, 7, 8, 9) indicate that, after adjustment for repeated measurements, none of the variables we examined had a significant influence on the production of an H1N1 positive aerosol (p<0.05) (Table 6). The value of the estimated odds ratio (OR) for the likelihood of producing PCR positive air samples in particle sizes ranges 0.86–7.3 µm in the presence of a WHO (2009) AGP was 4.31 (0.83–22.5). The categorical variable for WHO (2009) AGP was itself not significant.

**Table 7:** Analysis of air samples using the WHO (2007) AGP definitions.

Characteristic	All repeats (n = 99)		H1N1 positive aerosol samples^a^ (n = 14; collected from 10 patients)			A		B	
	n	%	n (n unique patients^b^)		Median copy no./l (inter- quartile range)	OR (95% CI)	p value	IRR (95% CI)	p value
AGP (2007)									
Baseline (no AGP)	56	57	5 (5)		9,862 (5,518–9,963)	referent		referent	
Bronchoscopy	3	3	2 (2)		111,702 (9,745–213,659)	36.8 (1.04–1302)		3.84 (0.52–28.1)	
High frequency oscillatory ventilation	6	6	0			–		–	
Intubation and related procedures	20	20	3 (2)		2,838 (1,543–20,614)	2.11 (0.34–13.2)		0.32 (0.07–1.48)	
Nebulisation	3	3	2 (2)		1,262 (671–1,852)	41.1 (1.20–1404)		0.05 (0.01–0.21)	
Non invasive ventilation	11	11	2 (2)		9,763 (7,913–11,613)	2.66 (0.30–24.0)	0.149	0.33 (0.07–1.60)	<0.001
AGP (2007) defined									
No						referent		referent	
Yes						4.25 (0.84–21.4)	0.079	1.04 (0.17–6.44)	0.964

a Sample considered positive if aerosol <7.3 µm indicated the present of H1N1.

b n unique patients refers to the number of patients these samples were taken from.

Column A: Results from univariable logistic regression models examining potential risk factors for production of H1N1 positive aerosol. Sample considered positive if aerosol <7.3 µm indicated the presence of H1N1. Estimates adjusted for repeated measurements.

Column B: Results from univariable negative binomial regression models examining potential risk factors for copy number per litre per minute given H1N1 positive aerosol.

A hyphen (-) indicates no positive samples in category. Referent refers to the category of an ordinal or nominal variable against which other categories are compared in the regression model and for which *no* dummy variable is included in the regression model.

**Table 8:** Analysis of the air samples using the WHO (2009) AGP definitions and including chest physiotherapy.

Characteristic	All repeats (n = 99)		H1N1 positive aerosol samples^a^ (n = 14; collectedfrom 10 patients)		A		B	
	n	%	n (n unique patients^b^)	Median copy no./l (inter- quartile range)	OR (95% CI)	p value	IRR (95% CI)	p value
AGP (2009) with Chest Physiotherapy								
Baseline (no AGP)	66	67	6 (5)	8,888 (5,518–11,613)	referent		referent	
Bronchoscopy	3	3	2 (2)	111,702 (9,745–213,659)	43.8 (1.06–1809)		4.37 (0.60–32.0)	
Lower respiratory tract specimen collection	1	1	0		–		–	
Intubation & related procedures	5	5	1 (1)	2,838 (2,838–2,838)	2.71 (0.15–49.1)		0.12 (0.03–0.50)	
Respiratory/airway suction	14	14	3 (2)	1,852 (1,543–20,614)	4.11 (0.50–34.0)		0.35 (0.07–1.70)	
Chest physiotherapy	10	10	2 (1)	5,317 (671–9,963)	3.06 (0.28–33.3)	0.449	0.23 (0.06–0.93)	not available
AGP (2009) defined including chest physiotherapy								
No					referent		referent	
Yes					4.36 (0.91–20.9)	0.065	1.30 (0.20–8.58)	0.787

a Sample considered positive if aerosol <7.3 µm indicated the present of H1N1.

b n unique patients refers to the number of patients these samples were taken from.

Column A: Results from univariable logistic regression models examining potential risk factors for production of H1N1 positive aerosol. Sample considered positive if aerosol <7.3 µm indicated the presence of H1N1. Estimates adjusted for repeated measurements.

Column B: Results from univariable negative binomial regression models examining potential risk factors for copy number per litre per minute given H1N1 positive aerosol.

A hyphen (-) indicates no positive samples in category. Referent refers to the category of an ordinal or nominal variable against which other categories are compared in the regression model and for which *no* dummy variable is included in the regression model.

**Table 9:** Analysis of the air samples using the WHO (2007) AGP definitions and including chest physiotherapy.

Characteristic	All repeats (n = 99)		H1N1 positive aerosol samples^a^ (n = 14; collected from10 patients)		A		B	
	n	%	n (n unique patients^b^)	Median copy no./l (inter- quartile range)	OR (95% CI)	p value	IRR (95% CI)	p value
AGP 2007 with Chest Physiotherapy								
Baseline (no AGP)	48	48	4 (4)	7,690 (3,414–56,967)	referent		referent	
Bronchoscopy	3	3	2 (2)	111,702 (9,745–213,659)	41.88 (1.06–1658)		3.33 (0.43–25.9)	
High frequency oscillatory ventilation	6	6	0		–		–	
Intubation and related procedures	20	20	3 (2)	2,838 (1,543–20,614)	2.32 (0.34–15.68)		0.8 (0.06–1.39)	
Nebulisation	3	3	2 (2)	1,262 (671–1,852)	48.4 (1.22–1918)		0.04 (0.01–0.21)	
Non invasive ventilation	11	11	2 (2)	9,763 (7,913–11,613)	2.92 (0.30–28.3)		0.28 (0.05–1.53)	
Chest physiotherapy	8	8	1 (1)	9,963 (9,963–9,963)	1.94 (0.11–33.5)	0.352	0.33 (0.08–1.45)	not available
AGP 2007 defined including chest physiotherapy								
No					Referent		referent	
Yes					3.91 (0.78–19.6)	0.098	0.85 (0.12–5.75)	0.865

a Sample considered positive if aerosol <7.3 µm indicated the present of H1N1.

b n unique patients refers to the number of patients these samples were taken from.

Column A: Results from univariable logistic regression models examining potential risk factors for production of H1N1 positive aerosol. Sample considered positive if aerosol <7.3 µm indicated the presence of H1N1. Estimates adjusted for repeated measurements.

Column B: Results from univariable negative binomial regression models examining potential risk factors for copy number per litre per minute given H1N1 positive aerosol.

A hyphen (-) indicates no positive samples in category. Referent refers to the category of an ordinal or nominal variable against which other categories are compared in the regression model and for which *no* dummy variable is included in the regression model.

Subject-level random effects obtained from the null logistic model indicate that one individual in the study has a significantly higher propensity than average to produce a positive sample, and that others have a raised propensity. This, coupled with the lack of significant relationships given in Tables 4, 5, 6, 7, 8, 9, suggests that propensity to produce a positive sample is more related to unmeasured individual-level attributes than demographic or other measured characteristics. The variation caused by the unmeasured attributes is high as the subject-level random effects have wide confidence intervals. The Variance Partition Coefficient (VPC) for the null logistic model indicates that only 31% of the total variance in the propensity to produce a positive sample is due to differences between individuals, for example in age or hospital location, and hence 69% is due to differences within individuals, for example, stage in infection. Unfortunately we do not have enough data to analyse an individual through time to investigate this further.

Results from the negative binomial regression are also given in Tables 4, 5, 6, 7, 8, 9. Copy number per litre is significantly positively associated with cases aged 50–60 years (incident rate ratio [IRR] 8.85 compared to referent, (95% CI: 4.14–18.9)) and cases from hospital location no. 2 (IRR 6.21(2.49–15.5)). It is significantly negatively associated with days since diagnosis (IRR 0.84 (0.79–0.90)); days since last positive sample (IRR 0.84 (0.78–0.90)); and relative humidity (IRR 0.95 (0.91–0.98)). However, WHO (2009) AGP categorisation itself was found to have no association with copy number, suggesting that some AGPs are associated with high copy numbers and others are associated with low copy numbers, as revealed by the IRR estimates for the various WHO (2009) AGP categories. These range from 0.15 to 5.41 (Table 6) but are, however, inconclusive because of the low numbers in each category. Our analysis of the influences on virus quantity in an aerosol is likely to be subject to confounding because of the low number of positive samples, and possible correlation between risk factors.

When the data are analysed with regard to the WHO (2007) AGP definitions there was a broad agreement with the results detailed above (Table 7). We also included procedures relating to chest physiotherapy in the analysis against AGP (2009) and AGP (2007) (Tables 8 & 9). The results suggest that chest physiotherapy is associated with an increased probability of aerosol production if included in the WHO (2009) AGP model (OR 3.06 (0.28–33.3)), but that aerosol titre is lower than baseline samples (IRR 0.23 (0.06–0.93)). To further establish the relationship between different AGPs the data were modelled to determine a hierarchy of procedures in terms of the potential risk of generating infectious aerosol (Table 10). We found that whilst most AGPs are associated with a higher probability of sampling an H1N1 positive aerosol they are also, with the exception of bronchoscopy, associated with lower copy numbers (l/min) than background samples and this lessens their overall risk (Tables 6, 7, 8, 9). Only bronchoscopy and respiratory/airway suctioning were found to have a risk greater than that encountered in the baseline (background) samples.

**Table 10:** Risk summary stratification table.

AGP definition	Sample size (number of repeats)	Number of H1N1 positive samples	Modelled probability of sampling H1N1 positive aerosol (assumes subject effect = 0)^a^	Modelled copy number (litres/min)^b^	Risk (defined as expected value of copy number, equal to modelled probability * modelled copy number)
Bronchoscopy (2009)	3	2	0.684	9,986	6,829
Bronchoscopy (2007)	3	2	0.659	9,986	6,579
Respiratory/airway suctioning (2009)	14	3	0.169	800	135
Baseline(2007)	48	4	0.044	3,003	132
Baseline (2009)	66	6	0.047	2,285	108
Non invasive ventilation (2007)	11	2	0.119	849	101
Nebulisation (2007)	3	2	0.691	126	87
Chest physiotherapy (2007)	8	1	0.082	996	82
Intubation (2007)	20	3	0.097	833	81
Chest physiotherapy (2009)	10	2	0.131	532	70
Intubation (2009)	5	1	0.118	284	33
Lower respiratory tract specimen (2009)	1	0	0.000	0	0
High frequency oscillatory ventilation (2007)	6	0	0.000	0	0

a Probabilities obtained from univariable logistic regression models examining potential risk factors for production of H1N1 positive aerosol. Sample considered positive if aerosol <7.3 µm indicated the presence of H1N1. Estimates adjusted for repeated measurements.

b Estimates obtained from univariable negative binomial regression models examining potential risk factors for copy number per litre per minute given H1N1 positive aerosol.

## Discussion

Evidence regarding the role of AGPs in the transmission of influenza in the hospital setting is limited. [5] The results from Table 2 indicate that smaller particles are preferentially produced during AGP procedures compared to baseline samples. However the total level of RNA recovered is higher during baseline samples, i.e. large particles containing higher quantities of RNA vs smaller particles containing smaller quantities of RNA. Due to the limitations of the sampling method it is impossible to quantify the total number of particles at each particle size – we only have an indication of the quantity of RNA collected in each particle size range. One might speculate (as others have done before) that a dispersed aerosol with a low concentration of influenza containing small particles might potentially be more of a transmission and morbidity risk than a few large particles containing significantly more total influenza. [5], [19].

This study detected influenza virus RNA in 14/99 (14.1%) air-sampling windows in particles 0.86–7.3 µm from 10/39 (25.6%) patients in broad agreement with reports from different health care settings [20], [21], [22], [23]. Of particular note is the fact that 19.6% of air specimens taken in the presence of a 2009 defined AGP (AGP 2009) revealed virus RNA infractions 0.86–7.3 µm, compared with only 7.9% in non-AGP (2009) settings (unadjusted OR = 2.84 (95% CI: 1.11–7.24) adjusted OR = 4.31 (0.83–22.5)).

Data were analysed for the propensity of an air-sampling event to produce a positive air sample in the 0.86–7.3 µM aerodynamic size range. None of the variables investigated in this study significantly increased the probability of obtaining a positive air sample for either the 2007 or 2009 analysis. However, there will be variability in the potential for the air sampler to detect positive air samples due to a number of factors including room ventilations patterns, the air particles’ initial velocities, temperature, relative humidity, and distance from source [24]. Even with this significant level of uncertainty and the low sample number we estimated an adjusted OR of 4.31 (0.83–22.5) for WHO (2009) AGP vs non WHO (2009) AGP settings (p = 0.083). Taken together with an unadjusted OR of 2.84, and in the context of the small study size, these findings suggest a true effect i.e. that AGPs as currently defined by 2009 WHO definitions increase the likelihood of generating infectious aerosols to the extent that this may be relevant in a clinical setting. However, we have almost certainly encountered a Type II statistical error due to small numbers. Further data are therefore required.

Analysis of the specific procedures shows that bronchoscopy is associated with the greatest probability of aerosol production (OR = 43.8(1.06–1809)), but both intubation and related procedures (OR = 2.71 (0.15–49.1)), respiratory/airway suction (OR = 4.11 (0.50–34.0)) and chest physiotherapy (OR = 3.06 (0.28–33.3)) also show increased probability, although none of these results attained statistical significance.

The quantity of viral RNA is known to vary both between patients and within individual patients during the time-course of their illness. Patients early in the course of infection excrete higher titres of virus and thus might generate aerosols containing more viral RNA. [25] In our study only 31% of the total variance in the propensity to produce a positive sample is due to differences between individuals, and 69% is due to differences within individuals. Unfortunately, we did not have ethical committee approval to obtain respiratory tract specimens from patients; thus although we can determine H1N1 diagnostic status from specimens taken by the attending physician (the basis of recruitment into this study), we cannot correlate our air sample findings against patient virus concentrations. Many other studies have also noted high variability in the number of virus particles expelled by subjects infected with respiratory pathogens therefore this work is consistent with previous studies. [22], [26], [27], [28], [29].

The influenza RNA loading of a positive air sample is likely to be influenced by the source of the aerosol (i.e. the patient and the location within the patient where the aerosol is produced). In a recent paper by Johnson et al., 2011, it has been suggested that aerosols of different particle sizes can be produced from different areas of the respiratory tract when different activities are performed, such as coughing, breathing and talking [30]. Although not investigated in this study the results suggest that the aerosols detected in our study could have originated from different parts of the respiratory tract, which could account for the wide range of viral titres picked up. If the viral loading differs along the respiratory tract and between individuals, and the different procedures have differing abilities to produce an aerosol, then a wide range of viral titres would be expected. However it is also true that variability in the data can be caused by differences in impinger proximity, orientation, directional air flows, human activity (producing turbulent air conditions) and ventilation levels between sampling sites.

The results indicate (although small sample size prohibits firm conclusions) that performance of a bronchoscopy increases the viral copy number per litre in positive air samples by a factor of 4.37 (CI = 0.60–32.0). Intubation and related procedures and respiratory/airway suctioning show decreased IRRs (0.12 (0.03–0.5) and 0.35 (0.07–1.70), respectively). This is the first evidence to suggest that some AGPs (e.g. bronchoscopy) may confer a greater risk of transmission to HCWs than others.

In order to further understand the risk hierarchy, titre and probability of a positive sample have been used to construct a risk summary stratification table (Table 10). This table has been produced simply as a method to summarise the results and modeled numbers are purely an indication of overall risk. However, this table shows that when probability of a positive air sample and the titre of an air sample are used to define risk, only bronchoscopy and the 2009 defined respiratory/airway suctioning carry a greater risk than the baseline samples. Positive baseline samples may be produced from residual aerosols left from previous AGPs, by some other unknown low level AGP, or by other H1N1 positive individuals coughing and sneezing in cohort areas.

The copy number of influenza virus recovered from a positive air sample is strongly influenced by hospital location (p<0.001), age range (p<0.001), day since diagnosis (p<0.001), days since last positive diagnostic sample (p<0.001), air sample volume (p<0.001) and relative humidity (p = 0.006).

Hospital location number 2 had significantly higher viral concentrations recovered from positive air samples potentially because this hospital is a tertiary referral centre for respiratory illnesses which provided extracorporeal membrane oxygenation (ECMO) for adults during the H1N1 outbreak. In addition, most bronchoscopies were performed at this location and this hospital contained the patient with a higher than average propensity to produce a positive sample.

Age was shown to have a significant effect with patients aged between 50–60 years having the highest rate of H1N1 aerosol generation in contrast to what would be expected from previous studies [31], [32]. This apparent age-related effect could be due to an outlying result; the patient with the significantly higher propensity than average to produce a positive air sample was in this group. A negative association between the copy number recovered and the number of days between diagnosis and sampling was found as would be expected. The significance of the relationship between high relative humidity and lower recovery of viral RNA correlated with previous studies [33].

### Limitations

This study has several limitations. Firstly, we did not perform a biological assay to quantify the viability and infectious potential of this viral material. Although the influenza virus is capable of surviving in air in the indoor environment there are no data regarding the stability of pandemic H1N1 (2009) in aerosols. The successful culture of viable viruses from environmental samples is technically difficult and thus relatively insensitive as a bioassay of infectivity [21], [22]. However, we believe that the detection of virus RNA in an aerosol within seconds of its generation, favours it being derived from viable viruses.

Secondly, even if an aerosol of the appropriate concentration does reach a health care worker and is inhaled, inhalation does not necessarily equate to infection. Infection depends upon infectious dose, route of inhalation (nose or mouth), tidal volume, breathing rate, timing and underlying susceptibility (immune status) [34]. It also depends upon particle size, for a particle to enter the distal lung it needs to be <5 µm for >10% deposition [35]. As we sampled in a wide range of different hospitals and it was very difficult to establish and get information about ventilation rates and patterns between hospitals and within different areas of the same hospital. In general isolation rooms had higher ventilation rates than ward cohort areas and in some cohort areas the opening of windows was the only form of ventilation evident. Obviously it was impossible to investigate fully all the areas we sampled due to time and logistical constraints in these busy environments. This study, therefore, can only give an indication of the levels of influenza RNA which a health-care worker could be exposed to.

Thirdly, estimating the aerosol infectious dose from previous studies is difficult due to the lack of studies in this area for health and ethical reasons [19], [36]. However, Alford *et al.,* (1967) found that the aerosol infectious dose of influenza A2/Bethesda/10/63 was between 0.6–3 TCID_50,_ which equates roughly to 0.6–3 virus particles according to Teunis *et al.,* (2010). [19], [37] The potential exposure of HCW during AGPs can be roughly calculated using the aerosol concentration, breathing rate and exposure time. If we assume a female HCW breathing rate of 15 l/min during an AGP lasting 15 minutes then 225 litres of air will be inhaled during the procedure. [38] The modelled concentration of viral copy numbers found during the bronchoscopy positive air samples was 9,986 copies/litre/minute, suggesting that expected exposure would be 2246850 virus copies. This exposure is significantly greater than the 0.6–3 TCID_50_ aerosol infectious dose reported by Alford *et al.,* in (1967). The study also shows that significant exposure to H1N1 aerosols (modelled copy number 2,285 copies/litre/minute) occurs in the absence of a recognized AGP in the vicinity. This study and that of Killingley *et al.,* suggest that exposure to aerosols of influenza may regularly occur during epidemics not just in ICUs or hospitals but in many other environments [20].

Fourthly, the May impinger does not collect particles <0.86 µm aerodynamic particle size, several studies have reported finding influenza RNA in air particles <1 µm, thus it is possible that some of the aerosolized RNA was missed [22], [23].

Finally, this was a large and complex study involving collaborations between the laboratory, sampling teams and busy clinicians from five hospitals. Due to the limited resources, lack of time and ethical constraints full clinical information on patients was not available. It is likely that the patients will represent a full spectrum of clinical presentations. Some patients would be in the early course of the infection while others had been infected for weeks, therefore, no information on viral load immediately prior to air sampling was available. In addition, there are numerous technical difficulties associated with the recovery of influenza from the air with the potential that many additional positive air samples have been missed due to the reasons previously discussed.

Notwithstanding the constraints imposed by small numbers, our data indicate that some AGPs as defined by WHO in 2009 do appear to be related to an increased likelihood of viral aerosol generation. The data are particularly clear for bronchoscopy and respiratory/airway suctioning. We conclude that AGPs have the potential to pose a threat of infection transmission to health care workers and that until further data are available, UK and WHO infection control policies for the use of high-filtration face pieces (respectively FFP3 and N95 respirators) during the performance of AGPs remain appropriate and should continue to be practiced according to local guidelines until more data are available.
